# Segmented Versus Global Optimization of Intraocular Lens Constants Across Axial Length: A Multi-Lens, Multi-Formula Comparison Using a Disjoint Training Dataset

**DOI:** 10.3390/diagnostics16172794

**Published:** 2026-08-30

**Authors:** Achim Langenbucher, Nóra Szentmáry, Alan Cayless, Peter Hoffmann, Kamran M. Riaz, Jascha Armin Wendelstein

**Affiliations:** 1 Department of Experimental Ophthalmology, Saarland University, 66424 Homburg/Saar, Germany; nszentmary@gmail.com (N.S.); wendelsteinjascha@gmail.com (J.A.W.); 2 Department of Ophthalmology, Semmelweis University, 1085 Budapest, Hungary; 3 School of Physical Sciences, The Open University, Milton Keynes MK7 6AA, UK; alan.cayless@gmail.com; 4 Augen- und Laserklinik Castrop-Rauxel, 44575 Castrop-Rauxel, Germany; 5 Dean McGee Eye Institute, University of Oklahoma Health Sciences Center, Oklahoma City, OK 73104, USA; kamran-riaz@dmei.org; 6 Department of Ophthalmology, LMU University Clinic, 80336 Munich, Germany

**Keywords:** intraocular lens power calculation, axial length, segmented regression, changepoint analysis, lens constant optimization, refractive prediction error, bootstrap confidence interval, biometry

## Abstract

**Background/Objectives:** Intraocular lens (IOL) constants are conventionally optimized globally across an entire calibration dataset, assuming that systematic prediction error is independent of axial length (AL). This assumption is known to fail in short and long eyes. We evaluated whether AL-segmented constant optimization reduces refractive prediction error, whether the benefit depends on formula family, and whether changepoints discovered in one population transfer to a disjoint one. **Methods:** In a pooled 6451-eye training dataset (6 IOL models), the corrected Akaike Information Criterion selected the optimal number (0–4) and positions of AL changepoints for six formulas (classic three-constant Haigis, new-Haigis H1/H2, SRK/T, Hoffer Q, Holladay 1), enforcing ≥40 eyes and ≥2.0 mm per segment. Locked changepoints were applied to two disjoint single-lens test subgroups (Vivinex, n = 887; SA60AT, n = 821; three-center retrospective cohort) and independently re-discovered within each. Root-mean-square prediction error (RMSE) reduction was assessed by bootstrap confidence interval, Diebold–Mariano test, and Wilcoxon signed-rank test. **Results:** Segmentation reduced training RMSE for all six formulas, particularly for Hoffer Q (−3.9%) and Holladay 1 (−2.8%; confidence intervals excluding zero, Diebold–Mariano *p* < 0.0001). With locked changepoints, both formulas again showed the largest test-dataset improvements (3.0–6.3%; confidence intervals excluding zero in all four lens–workflow combinations), followed by SRK/T (0.9–1.3%, up to 2.5% with independently discovered changepoints). Haigis-family formulas showed smaller reductions (0.2–1.6%) with confidence intervals including zero in several combinations. **Conclusions:** AL-segmented optimization benefits Hoffer Q and Holladay 1 most robustly—formulas lacking a direct anterior chamber depth predictor—with a smaller benefit for SRK/T and an inconsistent benefit for Haigis-family formulas. Changepoints from a pooled training population transfer usefully, though not optimally, to individual lens models.

## 1. Introduction

Accurate prediction of postoperative refraction after cataract surgery depends on the precision of the intraocular lens (IOL) power formula and the correctness of its constant, which calibrates the formula to a specific lens model and surgical setting [1,2]. Global constant optimization—zeroing the mean prediction error across a calibration dataset—is the established approach [2] and is the standard used by public registries such as IOLCon. It assumes, however, that the systematic component of prediction error is independent of axial length (AL). This assumption is well known to fail: vergence-based formulas predict effective lens position from AL through nonlinear relationships calibrated on historical populations, and short or long eyes deviate systematically from these relationships in ways that a single global constant cannot fully correct.

The problem manifests differently at opposite ends of the AL distribution. In long eyes, several widely used formulas overestimate effective lens position, leading to hyperopic surprises. Wang and Koch documented this specifically for Holladay 1 and SRK/T and proposed threshold-based AL adjustments as a correction [3,4]. In short eyes, Kenny et al. showed that the optimized constant shifts with AL in a way that degrades prediction accuracy for very short eyes relative to the normal AL range, and that re-deriving constants separately for short AL segments reduced prediction error beyond what artificial-intelligence formulas alone could achieve [5]. However, they also showed that segment sample sizes must be carefully managed—too few eyes per segment yields unstable constants—and a subsequent exchange specifically cautioned against zeroing the mean error in small or atypical AL subgroups [6].

A complementary question concerns what the optimized constant actually encodes. Langenbucher et al. showed that when constants are optimized across a heterogeneous, multi-lens or multicenter population, between-lens and between-center differences are absorbed into the constant in a way that can mask AL-dependent structure, and that the gradient of the optimized constant with respect to AL is formula-dependent and nonzero for all classical formulas, implying that a single global constant is always a compromise across the AL range [7].

Despite this background, no study has systematically compared AL-segmented constant optimization across multiple formula families, multiple IOL models, or with a rigorous statistical framework that distinguishes a genuine reduction in prediction error from sampling noise. Furthermore, existing analyses discover changepoints from the same data used to evaluate them (in-sample), without testing whether the identified changepoint structure generalizes to a disjoint population. A second unaddressed question is how many changepoints should be permitted: too many segments overfit, too few miss real structure.

Here we present a framework for AL-segmented IOL constant optimization that determines both the number and position of changepoints automatically using information-theoretic model selection [8,9,10], enforces minimum segment-size and segment-width constraints to prevent degenerate solutions, and tests changepoint transferability by applying training-dataset changepoints to a completely disjoint test cohort. We evaluate six vergence-based formulas across six IOL models, and report bootstrap confidence intervals and formal hypothesis tests alongside RMSE reductions so that statistical significance can be assessed alongside clinical magnitude.

## 2. Materials and Methods

### 2.1. Datasets

This retrospective comparative study used two fully disjoint datasets. The training dataset comprised 6451 eyes drawn from a pooled, multi-lens registry containing six monofocal IOL models (Vivinex, Hoya Surgical Optics; SA60AT, SN60WF and Clareon, all Alcon; ZCB00, Johnson & Johnson Vision; MX60, Bausch + Lomb); it was used exclusively for changepoint discovery and was never used to evaluate segmentation benefit. The test dataset comprised 3183 eyes from three clinical centers, containing the same six IOL models; it was used for evaluation only. The two most numerous test-dataset models, Vivinex (n = 887) and SA60AT (n = 821), were analyzed as individual lens subgroups. No eyes were shared between the training and test datasets.

Both datasets contained preoperative AL, external anterior chamber depth (ACD, measured from corneal vertex to lens), corneal radii in the flat and steep meridians, implanted IOL power, and postoperative spherical equivalent refraction (SEQ). Eyes were excluded if AL fell outside 15–40 mm, ACD outside 1–6 mm, corneal radii outside 5–12 mm, IOL power outside 0–50 D, or if SEQ was missing (four test-dataset eyes, 0.1%).

### 2.2. Formulas and Axial Length Correction

Six vergence-based formulas were evaluated: the classic three-constant Haigis formula (a0, a1, a2) [1]; two single-constant new-Haigis variants, denoted H1 (constant acting on implanted IOL power) and H2 (constant acting on effective lens position offset) [11]; SRK/T (A-constant) [12]; Hoffer Q (personalized ACD, pACD) [13]; and Holladay 1 (surgeon factor, SF) [14]. For all formulas, the manufacturer- or literature-recommended constant served as the unoptimized preset reference.

For the new-Haigis H1/H2 variants, a corrected AL (ALc) was computed per eye using a sum-of-segments type linear correction dependent on lens thickness (LT): ALc = 1.23854 + 0.95855 × AL − 0.05467 × LT, applied to eyes with a measured LT within a plausible range (2.5–7.0 mm). For eyes lacking a measured or plausible LT—2941 of 6451 training eyes (45.6%) and 0 of 3179 test eyes—an LT-independent linear approximation was substituted: ALc = 23.7 + 0.96 × (AL − 23.7). This per-eye fallback allowed every eye to contribute to formula optimization regardless of LT availability. All other formulas used raw, uncorrected AL, and the AL axis used for segmentation was always the raw AL.

### 2.3. Global and Segmented Constant Optimization

For each formula, two models were fitted to each dataset: a *global* model, in which a single constant (or constant vector, for the classic Haigis formula) was optimized across all AL values; and a segmented model, in which the AL range was divided into segments by one or more changepoints and an independent constant was optimized within each segment. Both models minimized root-mean-square prediction error (RMSE) using the Nelder-Mead simplex method with multiple starting points to reduce the risk of convergence to a local minimum. RMSE minimization was preferred over zeroing the mean prediction error (the convention for single-constant offset optimization) because the segment-specific constants act nonlinearly through effective lens position; consequently, a constant minimizing RMSE generally produces a small but nonzero residual mean error within each segment, which is reported for transparency.

For a fixed number of changepoints, changepoint positions were searched in two stages: an exhaustive grid search over candidate positions for zero to two changepoints, and a sequential forward-addition procedure with local refinement for three and four changepoints, to avoid the combinatorial cost of exhaustive search at higher counts. Every candidate solution was subject to two constraints: a minimum of 40 eyes per segment and a minimum of 2.0 mm of AL span per segment. The 40-eye minimum reflects published guidance on minimum sample sizes for stable constant optimization [5]. Quantitatively, 40 eyes corresponds to approximately 13 observations per free parameter for the classic three-constant Haigis formula, the most demanding case in our set, and to 40 observations per parameter for the single-constant formulas; the threshold was therefore set by the most demanding formula so that a single common constraint could be applied across all six. The 2.0 mm minimum width constraint was imposed specifically to prevent collinearity in the classic Haigis three-constant optimization: within a narrow AL segment, AL and ACD vary little, so the a0, a1, and a2 terms become strongly correlated and the normal equation system becomes ill-conditioned, yielding unstable individual constants even where the fitted values remain reasonable. The width constraint therefore operates alongside the sample-size constraint rather than instead of it. When these constraints prevented placing the full requested number of changepoints, the search returned the largest achievable number.

### 2.4. Automatic Selection of Changepoint Number

The number of changepoints (zero to a prespecified maximum of four) was selected automatically for each formula using the corrected Akaike Information Criterion (AICc), which penalizes additional free parameters more heavily than the standard Akaike Information Criterion (AIC) and is preferred when the ratio of sample size to parameter count is moderate [9,10]:

*AICc* = *N* · *ln*(*RSS*/*N*) + 2*k* + 2*k*(*k* + 1)/(*N* − *k* − 1),

where RSS is the residual sum of squares, N is the number of eyes, and k is the number of free parameters (segment-specific formula constants plus changepoint positions). The AIC and the Bayesian Information Criterion (BIC) [8] were computed alongside AICc for comparison (Figure S3). The changepoint count minimizing AICc was selected as the final segmented model for each formula.

### 2.5. Analysis Workflows

Three analysis workflows were performed (Figure S1). **Workflow A**: all 6451 training-dataset eyes were used to discover, for each formula independently, the AICc-optimal changepoint number and positions. Because the training dataset pools six lens models with differing AL distributions, this workflow tests whether a changepoint structure derived from a heterogeneous, pooled population is useful when transferred to an individual lens model. **Workflow B**: the changepoint positions discovered in Workflow A were locked and applied, without re-optimization of position, to the Vivinex (n = 887) and SA60AT (n = 821) test subgroups; only the segment-specific constants were re-optimized within each locked segment. **Workflow C**: the changepoint number and positions were re-discovered independently within each single-lens test subgroup, by the same AICc procedure used in Workflow A, without reference to the training dataset or to Workflow B. Workflow C represents the benchmark of what segmentation can achieve when changepoints are derived from the lens-specific population itself, and allows direct comparison against the locked-changepoint approach of Workflow B.

### 2.6. Outcome Measures and Statistical Analysis

For each formula and model (preset, global, segmented), we report the mean prediction error (ME), standard deviation (SD), mean absolute error (MAE), and RMSE, following recommended reporting standards for IOL power calculation studies [2,15]. The primary outcome was RMSE, and the primary comparison metric was the relative RMSE reduction from global to segmented optimization.

For every formula and workflow, this reduction was supplemented by three statistical comparisons: (1) a bootstrap 95% confidence interval on the RMSE difference between global and segmented models, using 2000 resamples; (2) the Diebold–Mariano test applied to paired squared prediction errors; and (3) the Wilcoxon signed-rank test on paired absolute prediction errors. In each bootstrap replicate, the changepoint positions were held fixed at those selected by AICc from the original dataset, and only the eyes and the segment-specific constants were resampled and re-estimated; consequently, the confidence intervals capture uncertainty in the constants given fixed changepoints, but do not propagate uncertainty in the changepoint selection step itself. The Diebold–Mariano test, originally developed for autocorrelated time-series forecast errors, is applied here to cross-sectional prediction errors as a test of equal predictive accuracy on paired squared errors, without a temporal ordering assumption. A bootstrap confidence interval excluding zero together with a Diebold–Mariano *p*-value below 0.05 was taken as evidence that the segmented model’s RMSE improvement was unlikely to be attributable to sampling variability alone.

All analyses were performed in MATLAB R2025b (MathWorks, Natick, MA, USA).

### 2.7. Ethics and Use of Generative Artificial Intelligence

This study was conducted in accordance with the tenets of the Declaration of Helsinki. As a retrospective analysis of fully de-identified routine clinical data, ethical review and approval were waived, and institutional data-protection regulations were observed throughout. During the preparation of this work the authors used a large language model (Opus 4.8, Claude, Anthropic) to assist with drafting and editing the manuscript text. The study concept, the analysis platform, the choice of statistical methods, the interpretation of the results and all scientific conclusions are those of the authors. No data, numerical results or references were generated by the tool; all numerical values reported in this manuscript were produced by the authors’ own MATLAB analysis platform and verified against its saved output. After using this tool, the authors reviewed and edited the content as needed and take full responsibility for the content of the publication.

## 3. Results

### 3.1. Dataset Characteristics

After exclusion of four test-dataset eyes (0.1%) for physiologically implausible values, 3179 test eyes and 6451 training eyes were available for analysis. Lens thickness was available for 54.4% of training eyes and 99.9% of test eyes; the remaining training eyes used the per-eye LT-independent approximation for AL correction. Descriptive statistics for both datasets are presented in Table 1. Both datasets were dominated by eyes of normal axial length: 5754 training eyes (89.2%) and 2610 test eyes (82.0%) fell between 22.0 and 26.0 mm, whereas only 409 (6.3%) and 288 (4.5%) training eyes were shorter than 22.0 mm or longer than 26.0 mm, respectively. The corresponding proportions in the test dataset were 306 (9.6%) and 267 (8.4%). The full axial length distributions, with the discovered changepoint positions overlaid, are shown in Figure S5.

### 3.2. Training Dataset: Changepoint Discovery (Workflow A)

AICc-guided segmentation reduced RMSE for all six formulas in the training dataset (Table 2). The Haigis-family formulas (classic three-constant Haigis, new-Haigis H1, new-Haigis H2) showed small reductions in RMSE point estimates of 0.2% to 0.5%; although bootstrap confidence intervals excluded zero for all three, the Diebold–Mariano test statistics were markedly smaller than for Hoffer Q or Holladay 1. SRK/T showed a reduction of 1.0%, also with a confidence interval excluding zero. The corresponding shift in the distribution of absolute prediction error is shown in Figure S2.

**Figure 1:**
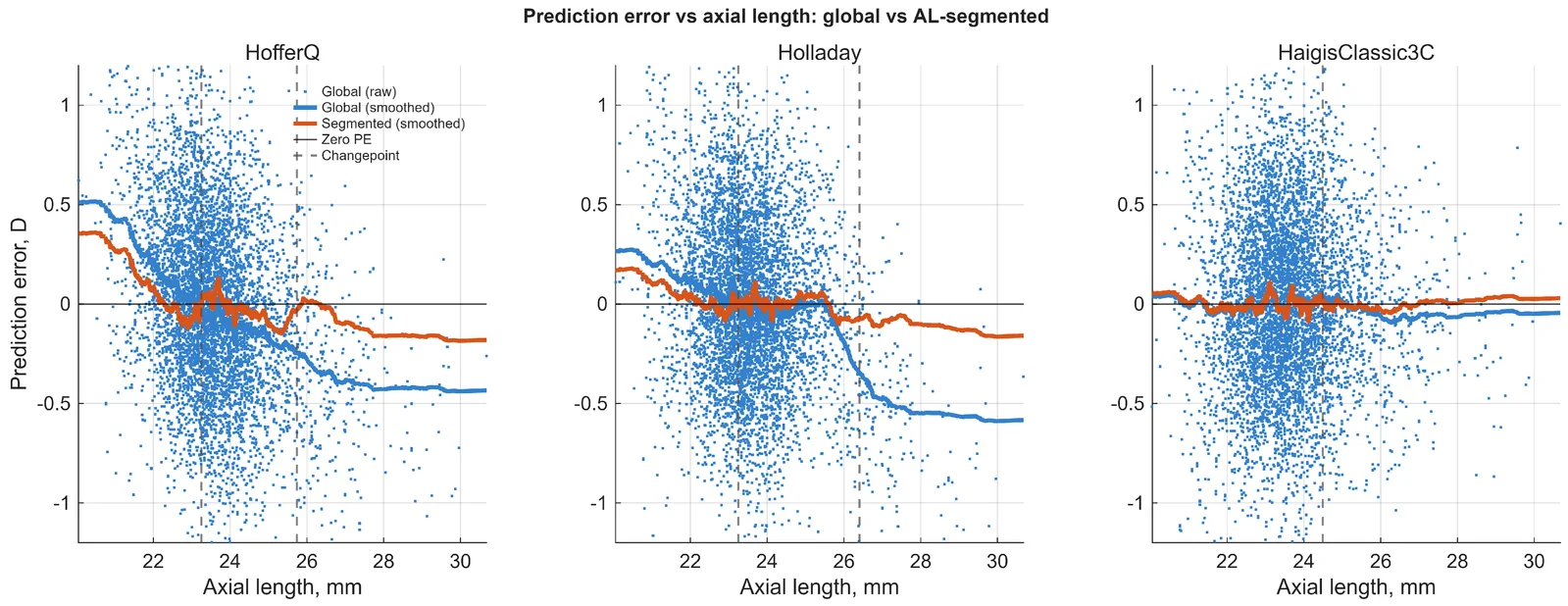
Prediction error (D) versus axial length (mm) for Hoffer Q, Holladay 1, and the classic three-constant Haigis formula in the training dataset. Each panel shows raw prediction errors for the global model, smoothed trend lines for the global and segmented models, and vertical dashed lines marking the AICc-selected changepoint positions. AL = axial length; D = diopters. The legend is placed in the upper right of each panel.

Hoffer Q and Holladay 1 showed the largest reductions: 3.9% (0.470 → 0.452 D, two changepoints at AL 23.25 and 25.74 mm) and 2.8% (0.446 → 0.433 D, changepoints at 23.25 and 26.41 mm), respectively (Figure 1). Bootstrap 95% confidence intervals excluded zero by a wide margin for both formulas (Hoffer Q: 0.0153 to 0.0215 D; Holladay 1: 0.0099 to 0.0151 D), with Diebold–Mariano *p*-values below 0.0001 for both. In absolute terms, these correspond to reductions of 0.018 D and 0.013 D, respectively.

### 3.3. Single-Lens Test Subgroups (Workflows B and C)

**Figure 2:**
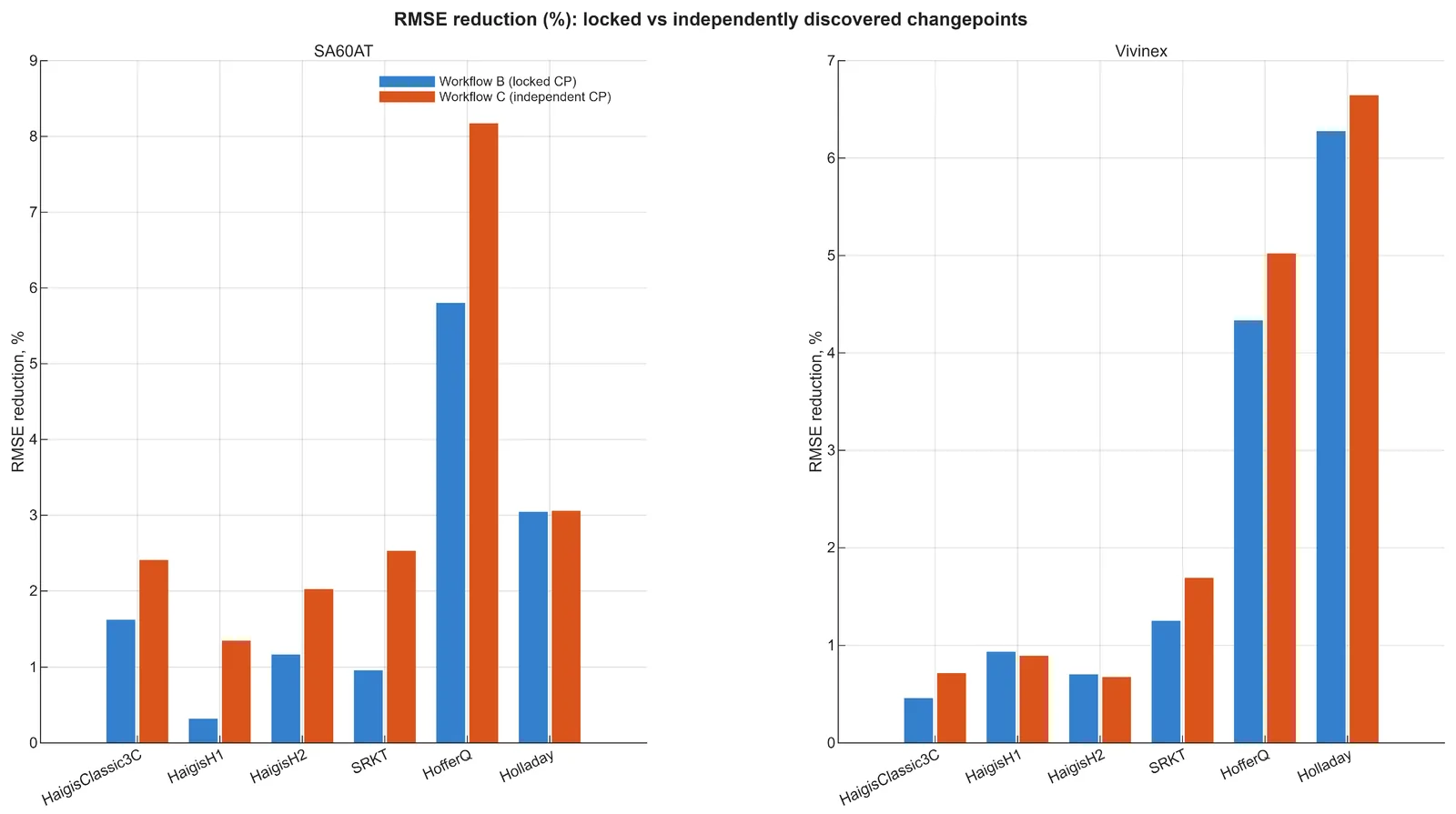
Relative RMSE reduction (%) for Workflow B (locked training-derived changepoints) versus Workflow C (independently discovered changepoints) by formula, shown separately for the Vivinex and SA60AT subgroups. Bars are annotated to indicate formula–lens combinations for which the bootstrap 95% confidence interval excluded zero. RMSE = root-mean-square prediction error.

When training-derived changepoints were locked and applied to the test subgroups (Workflow B), the same formula-dependent pattern held (Table 3, Figure 2). Hoffer Q and Holladay 1 showed RMSE reductions of 3.0% to 6.3% (0.014 to 0.027 D in absolute terms), each with a bootstrap confidence interval excluding zero and a Diebold–Mariano *p*-value below 0.001 in both lenses. SRK/T showed smaller reductions (0.9% to 1.3%) that nonetheless achieved statistical significance in both lenses, though with markedly smaller Diebold–Mariano test statistics (2.1 to 2.6) than Hoffer Q or Holladay 1 (3.7 to 4.9).

For the Haigis-family formulas the pattern was less consistent. Bootstrap confidence intervals included zero for new-Haigis H1 in both Vivinex workflows and in SA60AT under Workflow B, and for new-Haigis H2 and classic Haigis in Vivinex. Only in the SA60AT subgroup under Workflow C did the confidence intervals clearly exclude zero across the Haigis family. This indicates that for these three formulas the reduction in RMSE point estimates, while consistently positive, was not reliably distinguishable from sampling variability at the single-lens sample sizes available here, particularly in the Vivinex subgroup.

Independent changepoint discovery within each subgroup (Workflow C) selected different AL positions than Workflow B for every formula; none of the independently selected changepoint positions matched the training-derived positions. Despite this, Workflow C produced comparable or marginally larger RMSE reductions in every case: for Hoffer Q, 5.0% versus 4.3% in Vivinex and 8.2% versus 5.8% in SA60AT; for Holladay 1, 6.7% versus 6.3% in Vivinex and 3.1% versus 3.0% in SA60AT. Statistical significance patterns were broadly similar between the two workflows for all formulas. A paired comparison of global versus segmented prediction errors for Hoffer Q in Vivinex is shown in Figure S4.

### 3.4. Constraint Behavior and Segment Sample Sizes

The joint minimum-segment-size (40 eyes) and minimum-segment-width (2.0 mm) constraints prevented placing three or four changepoints for every formula in the training dataset; in each case the search returned the same two-changepoint solution already found at two changepoints, confirming that the constraints, rather than a genuine higher-order optimum, limited the achievable model complexity. AICc then selected one changepoint for the classic Haigis formula and two changepoints for the remaining five formulas (Figure S3). In the smaller single-lens subgroups, locking training-derived changepoints occasionally produced segments below the 40-eye minimum, the smallest being 16 eyes for new-Haigis H1 and H2 in Vivinex; constants derived for these segments are reported for completeness but should not be regarded as clinically actionable without external validation. To assess the influence of the 40-eye threshold itself, Workflow A discovery was repeated with minimum segment sizes of 30 and 50 eyes (Table S2). The AICc-selected number of changepoints was unchanged for all six formulas at both thresholds. Changepoint positions were essentially invariant for five formulas (maximum shift 0.01 to 0.07 mm relative to the 40-eye solution); only SRK/T showed an appreciable shift, its upper changepoint moving from 25.74 mm to 26.36 mm at a 50-eye minimum (maximum shift 0.62 mm).

## 4. Discussion

This study found that AL-segmented constant optimization reduced RMSE for all 6 evaluated formulas, using a disjoint two-dataset design that cleanly separates changepoint discovery from evaluation. The magnitude and statistical robustness of the benefit were, however, strongly formula-dependent. Hoffer Q and Holladay 1 showed reductions of 3% to 8%, with bootstrap confidence intervals excluding zero in every lens–workflow combination tested. SRK/T showed a smaller but consistently significant benefit. The Haigis-family formulas, which already incorporate ACD (classic Haigis) or a corrected AL (new-Haigis H1/H2), showed reductions in RMSE point estimates that were frequently not distinguishable from sampling variability at single-lens sample sizes.

This formula-dependence is mechanistically plausible. Formulas that predict effective lens position from AL alone, through fixed nonlinear terms calibrated on historical populations, retain residual AL-dependent error that segmentation directly removes. The classic Haigis formula incorporates ACD alongside AL as an explicit second predictor of effective lens position, substantially reducing the AL-dependent component of trend error; the new-Haigis H1 and H2 variants use a sum-of-segments type AL correction specifically intended to remove this nonlinearity before the formula is applied [7,11]. The intermediate position of SRK/T—a real but consistently smaller benefit than Hoffer Q or Holladay 1—is consistent with SRK/T’s own partial AL correction, a retinal thickness correction applied for AL beyond 24.2 mm, which may already remove part of the nonlinearity that segmentation would otherwise capture. Consistent with this interpretation, tuning of the Haigis constants has been shown to reduce AL-dependent trend error without any segmentation at all [16], indicating that a substantial part of the residual nonlinearity in this formula family is already accessible to constant optimization itself and is therefore not available for segmentation to remove.

A central methodological observation is that reporting RMSE point estimates alone would have misrepresented these results. Every Haigis-family point estimate was positive—segmentation never increased RMSE on average—yet the 95% confidence interval included zero in several lens–formula combinations, most consistently for Vivinex. Reporting reduction percentages without a corresponding measure of statistical support would have presented segmentation as a uniform, formula-independent benefit. Conversely, in the 6451-eye training dataset, reductions as small as 0.2% to 0.5% attained nominal statistical significance purely through large sample size, despite being well below any clinically meaningful threshold. Statistical significance and clinical relevance must therefore be assessed separately, and we suggest that future segmented-optimization studies report both the magnitude of reduction and a formal measure of statistical support.

Changepoints discovered in a pooled, heterogeneous training population transferred usefully to disjoint single-lens test subgroups. Workflow B, using locked training-derived changepoints, achieved reductions broadly comparable to the unconstrained benchmark of Workflow C for Hoffer Q and Holladay 1, even though none of the independently discovered changepoint positions matched the training-derived ones. This suggests that, for the formulas where segmentation is the dominant source of benefit, the presence of segmentation matters more than the precise changepoint position. It supports the practical use of pooled training data for changepoint discovery when lens-specific sample sizes are insufficient—provided segment sample sizes are monitored. Kenny et al. emphasized that a minimum sample size per AL segment is necessary for segmented constants to be reproducible [5], and a subsequent exchange cautioned specifically against zeroing the mean error in small or atypical AL subgroups [6]. The smallest segments observed in our locked-changepoint analysis (16 eyes) fall well below any reasonable stability threshold, underlining the importance of this check before clinical adoption.

Our approach differs conceptually from the Wang–Koch AL adjustment and its successors [3,4,17], which retain a single global constant but transform the AL value entered into the formula for eyes beyond a fixed threshold. The two approaches therefore intervene at different points in the calculation chain. An AL transformation modifies the input variable before it reaches the formula: the measured AL is replaced by an adjusted value, which then propagates through the formula’s unchanged AL-to-effective-lens-position mapping, so the shape of that mapping is preserved and only the point at which it is evaluated moves. Segmented constant optimization leaves the measured AL untouched and instead allows the constant—which enters the effective lens position prediction additively or multiplicatively depending on formula—to take different values in different AL ranges, in effect fitting a piecewise offset to the effective lens position prediction itself. A further practical difference is that an AL transformation applies a single fixed rule at a predetermined threshold, whereas segmentation derives both the number and the location of the breakpoints from the data, at the cost of requiring sufficient eyes within every segment. The substantially larger and more robust benefit we observed for Holladay 1—the same formula for which the original Wang–Koch adjustment was developed—suggests that these two approaches may address the same underlying nonlinearity from different directions. The magnitude of improvement observed for Hoffer Q and Holladay 1 is comparable to that reported for intraoperative aberrometry-based constant optimization [18], indicating that AL segmentation can offer clinically relevant gains without additional intraoperative measurement.

A methodological limitation of the discovery step is that changepoints and constants were estimated jointly from the same pooled, multi-lens training population, with between-lens optical differences absorbed into the residual error against which RMSE is minimized. An alternative hierarchical design—shared changepoint positions with lens-specific constants within each segment—would more cleanly disentangle genuine AL-dependent structure from between-lens heterogeneity, since a changepoint could in principle emerge in a pooled analysis simply because two lens models have different AL distributions and different optimal constants, rather than because of true within-lens AL-dependent nonlinearity. We did not implement this here because lens-specific constants estimated during training would partially pre-empt the transferability question that Workflows B and C are designed to test. We regard this hierarchical formulation as a worthwhile direction for future work, run as an additional labeled workflow rather than a replacement for the present design.

### 4.1. Pooled-Dataset Heterogeneity and Changepoint Validity

Because Workflow A discovers changepoints in a population that pools six IOL models, it is legitimate to ask whether some of the resulting breakpoints are artefacts of that pooling rather than reflections of within-lens AL-dependent error. The mechanism by which such an artefact could arise is straightforward: if two lens models have different optimal constants and occupy partly different regions of the AL distribution, the pooled residual will show a step at the axial length where the mixture proportion changes most sharply, and a changepoint search may localize that step even in the complete absence of within-lens nonlinearity. A hierarchical or mixed-effects formulation would address this directly, treating the changepoint positions as fixed effects shared across lens models while allowing the segment constants to vary as random effects by lens. We did not adopt that design here because estimating lens-specific constants during discovery would partially pre-empt the transferability question that Workflows B and C exist to answer, and we regard it as the natural next step rather than a substitute for the present analysis.

The available empirical evidence does not suggest that pooling systematically distorted the changepoint positions. Comparing the pooled Workflow A changepoints against those discovered independently within each single-lens subgroup (Workflow C) shows agreement in the selected number of changepoints in 9 of 12 formula–lens combinations, with a median absolute deviation in position of 0.59 mm across the 20 directly comparable changepoints (Table S1). Critically, the deviations show no directional tendency: 10 of 20 independently discovered changepoints lay at longer axial lengths than their pooled counterparts and 10 at shorter (two-sided sign test, *p* = 1.0). A systematic pooling bias would be expected to displace the lens-specific changepoints consistently in one direction; the absence of any such tendency is more consistent with estimation noise in moderately sized subgroups. The largest single deviation (2.91 mm, classic Haigis in SA60AT) arises where Workflow A selected one changepoint and Workflow C selected two, so only the first is comparable and the figure overstates the true disagreement.

### 4.2. Statistical Significance Versus Clinical Relevance

The separation between statistical and clinical significance deserves an explicit criterion rather than a general caution, and any such criterion must be stated in terms of the quantity actually being compared. A reduction in RMSE describes the error distribution of a population, not any individual prediction: lowering RMSE from 0.45 to 0.40 D does not move a single eye’s predicted refraction by 0.05 D, it narrows the distribution from which that eye’s error is drawn. We therefore set the threshold by asking what change in RMSE would visibly alter the proportion of eyes reaching the conventional ±0.50 D refractive target. For an approximately normal and approximately centered error distribution at the baseline RMSE of about 0.45 D observed here, a reduction of 0.05 D raises that proportion from roughly 73% to roughly 79%, or about five to six additional eyes per 100. The same figure is a little over one tenth of the baseline error dispersion, so it also marks the point at which the improvement becomes an appreciable fraction of the error it is intended to reduce rather than a marginal adjustment to it. We adopt 0.05 D on these grounds, while emphasizing that it is a convention adopted here for interpretation and not an established standard, and that the translation is approximate because prediction errors are neither exactly normal nor exactly centered.

Measured against this threshold, none of the reductions reported here is clinically meaningful at the level of the individual patient: the largest absolute improvement in any single-lens test subgroup was 0.027 D (Hoffer Q, SA60AT), with typical improvements between 0.005 and 0.027 D. The best-performing combinations nonetheless fall short of the threshold rather than being negligible: applying the same translation, the 0.027 D reduction for Hoffer Q in SA60AT corresponds to approximately three additional eyes per 100 within ±0.50 D, and the reduction for Holladay 1 in Vivinex to a similar figure. Axial length segmentation would therefore be unlikely to change the implanted power for an individual eye, and its value lies in a modest narrowing of the population error distribution rather than in the individual surgical decision. Two further consequences follow for how such results should be read. First, the width and position of a bootstrap confidence interval should be interpreted against a clinical threshold and not only against zero. The Hoffer Q training-dataset interval of 0.0153 to 0.0215 D excludes zero decisively while lying entirely below any clinically meaningful magnitude, and reporting only that it excludes zero would conceal precisely that fact. Second, because the Diebold–Mariano statistic scales with the square root of the sample size, a reduction of 0.2% attains *p* < 0.05 in the 6451-eye training dataset while an identical effect in an 800-eye subgroup would not. Statistical significance in the training dataset therefore reflects sample size at least as much as effect magnitude, and the pattern of significance across our workflows should be read with that asymmetry in mind.

Other limitations should be considered. This was a retrospective, non-randomized analysis; although changepoints were discovered in a disjoint training population, the segment-specific constants were fitted and evaluated on the same test-dataset eyes, so the reported test-dataset RMSE reductions retain some in-sample optimism with respect to those constants. Biometer type, surgeon, and surgical technique were not available as covariates. The maximum number of changepoints was fixed at four a priori, and the joint constraints meant that the three- and four-changepoint searches achieved no better than the two-changepoint solution, so we cannot exclude that finer segmentation would help in a larger dataset with relaxed constraints. Detailed single-lens analysis was restricted to the two most numerous IOL models. Lens thickness was unavailable for 45.6% of training-dataset eyes, necessitating the per-eye LT-independent approximation for the new-Haigis variants; the choice of AL conversion is itself not neutral, since different approaches yield systematically different corrected AL values [19]. We examined the consequences of this directly (Table S3). Eyes with and without a usable lens thickness measurement differed slightly but statistically significantly in axial length (23.642 ± 1.337 mm versus 23.558 ± 1.085 mm; difference 0.085 mm, *p* = 0.006), so LT availability is not strictly missing-at-random with respect to the segmentation axis, although a difference of 0.085 mm against a within-group standard deviation of approximately 1.2 mm is of no practical consequence and is itself an illustration of the significance-versus-relevance distinction discussed above. Repeating Workflow A discovery on the 3510 LT-available eyes alone left the changepoint positions of SRK/T and Holladay 1 essentially unchanged (shifts of 0.13 and 0.11 mm) but moved those of the Haigis family substantially: new-Haigis H1 shifted by 1.41 mm, new-Haigis H2 reduced to a single changepoint, and the classic Haigis formula no longer selected any segmentation at all. Hoffer Q, which uses raw AL, shifted by 0.81 mm, indicating that a component of this movement reflects the halving of the sample rather than lens thickness as such. The formulas most affected are nonetheless precisely those that consume lens thickness through the corrected AL, and their training-dataset changepoints must therefore be regarded as the least securely established of those we report. Test-dataset results, where LT was available for 99.9% of eyes, are not subject to this limitation. Finally, segmentation was performed only along AL; segmentation along implanted IOL power was not evaluated, and the constants derived here have not been externally validated in a further independent cohort.

## 5. Conclusions

Axial length-segmented optimization of IOL constants reduced refractive prediction error beyond global optimization across six vergence-based formulas, but the benefit was concentrated in, and statistically most robust for, Hoffer Q and Holladay 1—formulas that predict effective lens position from AL without a direct anterior chamber depth term. SRK/T showed a smaller but consistently significant benefit. For the Haigis-family formulas, which already incorporate ACD or a corrected AL, the segmentation benefit was frequently not statistically distinguishable from zero at single-lens sample sizes, despite consistently positive point estimates. Changepoints discovered in a disjoint, pooled, multi-lens training population transferred usefully—though not optimally—to individual lens subgroups, supporting the use of pooled training data for changepoint discovery when lens-specific data are insufficient. The absolute magnitude of these gains is nonetheless modest: the largest improvement in any single-lens subgroup was a 0.027 D reduction in RMSE, corresponding to approximately three additional eyes per 100 within ±0.50 D of the refractive target. Segmentation should therefore be understood as a population-level refinement of the prediction error distribution rather than as an intervention that will alter the lens chosen for an individual eye. Segment sample sizes should be verified before any resulting constant is adopted in clinical practice.

## Figures and Tables

**Table 1:** Descriptive statistics of the training and test datasets. Continuous variables are given as mean ± standard deviation, with median and interquartile range (IQR) additionally reported for axial length.

Variable	Training (n = 6451)	Test (n = 3183)
AL (mm)	23.60 ± 1.23	23.79 ± 1.60
AL, median (IQR), mm	23.48 (22.84–24.19)	23.63 (22.85–24.51)
ACD, external (mm)	3.11 ± 0.40	3.16 ± 0.41
Mean keratometry (D)	43.80 ± 1.47	43.76 ± 1.50
IOL power (D)	21.53 ± 3.26	21.28 ± 4.40
Postoperative SEQ (D)	−0.36 ± 0.66	−0.54 ± 0.83
LT available, n (%)	3510 (54.4)	3179 (99.9)
AL < 22.0 mm, n (%)	409 (6.3)	306 (9.6)
AL 22.0–26.0 mm, n (%)	5754 (89.2)	2610 (82.0)
AL > 26.0 mm, n (%)	288 (4.5)	267 (8.4)

ACD = anterior chamber depth; AL = axial length; D = diopters; IOL = intraocular lens; LT = lens thickness; SEQ = spherical equivalent refraction. Axial length strata follow the conventional short/normal/long classification.

**Table 2:** Training-dataset results: global versus axial length-segmented optimization (Workflow A, n = 6451).

Formula	Global RMSE (D)	Segmented RMSE (D)	Reduction (%)	Changepoints (mm)	95% CI on ΔRMSE	D-M *p*
Haigis 3C	0.4475	0.4467	0.2	24.49	0.00016, 0.00132	0.011
New-Haigis H1	0.4443	0.4422	0.5	22.94, 26.41	0.00104, 0.00312	<0.001
New-Haigis H2	0.4437	0.4425	0.3	22.94, 26.41	0.00040, 0.00203	0.002
SRK/T	0.4579	0.4535	1.0	23.25, 25.74	0.00290, 0.00594	<0.001
Hoffer Q	0.4702	0.4518	3.9 *	23.25, 25.74	0.01526, 0.02146	<0.0001 *
Holladay 1	0.4458	0.4334	2.8 *	23.25, 26.41	0.00993, 0.01514	<0.0001 *

* Denotes a statistically significant RMSE reduction (bootstrap confidence interval excluding zero and Diebold–Mariano *p* < 0.05). CI = bootstrap 95% confidence interval on the RMSE difference; D = diopters; D-M = Diebold–Mariano test; RMSE = root-mean-square prediction error. Where the minimum segment-size and segment-width constraints prevented placing the full requested number of changepoints, the largest achievable number is shown (Figure S3).

**Table 3:** Locked training-derived changepoints (Workflow B) versus independently discovered changepoints (Workflow C) in two single-lens test subgroups.

Formula	Lens	WF	Global RMSE (D)	Segmented RMSE (D)	Reduction (%)	CI Excludes 0	D-M *p*
Haigis 3C	Vivinex	B	0.3678	0.3661	0.5	No	0.152
	Vivinex	C	0.3678	0.3651	0.7	No	0.075
	SA60AT	B	0.4154	0.4087	1.6	Yes	0.005
	SA60AT	C	0.4154	0.4054	2.4	Yes	0.002
New-Haigis H1	Vivinex	B	0.3496	0.3463	0.9	No	0.059
	Vivinex	C	0.3496	0.3465	0.9	No	0.064
	SA60AT	B	0.4025	0.4012	0.3	No	0.208
	SA60AT	C	0.4025	0.3971	1.3	Yes	0.021
New-Haigis H2	Vivinex	B	0.3485	0.3460	0.7	No	0.102
	Vivinex	C	0.3485	0.3461	0.7	No	0.103
	SA60AT	B	0.4027	0.3980	1.2	Yes	0.030
	SA60AT	C	0.4027	0.3945	2.0	Yes	0.004
SRK/T	Vivinex	B	0.4387	0.4332	1.3	Yes	0.009
	Vivinex	C	0.4387	0.4313	1.7	Yes	0.002
	SA60AT	B	0.4868	0.4822	0.9	Yes	0.023
	SA60AT	C	0.4868	0.4745	2.5	Yes	0.001
Hoffer Q	Vivinex	B	0.4259	0.4075	4.3 *	Yes	<0.0001 *
	Vivinex	C	0.4259	0.4045	5.0 *	Yes	<0.0001 *
	SA60AT	B	0.4615	0.4347	5.8 *	Yes	<0.0001 *
	SA60AT	C	0.4615	0.4238	8.2 *	Yes	<0.0001 *
Holladay 1	Vivinex	B	0.4236	0.3970	6.3 *	Yes	<0.0001 *
	Vivinex	C	0.4236	0.3954	6.7 *	Yes	<0.0001 *
	SA60AT	B	0.4434	0.4299	3.0 *	Yes	<0.001 *
	SA60AT	C	0.4434	0.4298	3.1 *	Yes	0.001 *

* Denotes a statistically significant RMSE reduction (bootstrap confidence interval excluding zero and Diebold–Mariano *p* < 0.05). CI = bootstrap 95% confidence interval; D = diopters; D-M = Diebold–Mariano test; RMSE = root-mean-square prediction error; WF = workflow (B = locked training-derived changepoints; C = independently discovered changepoints).

## Data Availability

The minimal dataset supporting the reported results is available from the corresponding author on reasonable request. Patient-level biometry data cannot be shared publicly owing to data protection and privacy regulations.

## Supplementary Materials

The following supporting information can be downloaded at: https://www.mdpi.com/article/10.3390/diagnostics16172794/s1. Figure S1: Schematic diagram of the three analysis workflows; Figure S2: Empirical cumulative distribution function of absolute prediction error; Figure S3: AIC, AICc, and BIC model-selection profiles by changepoint number; Figure S4: Bland–Altman comparison of global versus segmented prediction error for Hoffer Q in the Vivinex subgroup; Figure S5: Axial length distributions of the training and test datasets with changepoint positions overlaid; Table S1: Workflow A versus Workflow C changepoint positions; Table S2: Sensitivity of changepoint discovery to the minimum segment size; Table S3: Sensitivity of changepoint discovery to lens thickness availability.

## Abbreviations

The following abbreviations are used in this manuscript:

ACD	Anterior chamber depth
AIC	Akaike Information Criterion
AICc	Corrected Akaike Information Criterion
AL	Axial length
BIC	Bayesian Information Criterion
CI	Confidence interval
D	Diopters
D-M	Diebold-Mariano
IOL	Intraocular lens
LT	Lens thickness
MAE	Mean absolute error
ME	Mean prediction error
RMSE	Root-mean-square prediction error
SEQ	Spherical equivalent refraction
