# Supplementary material for: Segmented Versus Global Optimization of Intraocular Lens Constants Across Axial Length: A Multi-Lens, Multi-Formula Comparison Using a Disjoint Training Dataset
Source: Diagnostics (Basel). 2026 Aug 30;16(17):2794. doi: 10.3390/diagnostics16172794 (PMC13565236; doi:10.3390/diagnostics16172794)
Supplement: Supplementary file 1 [file diagnostics-16-02794-s001.zip › diagnostics-4520903-supplementary.pdf]

Supplementary Materials

Segmented Versus Global Optimization of Intraocular Lens Constants Across Axial Length: A Multi-Lens, Multi-Formula Comparison Using a Disjoint Training Dataset

Achim Langenbucher, Nóra Szentmáry, Alan Cayless, Peter Hoffmann, Kamran Riaz and Jascha Armin Wendelstein

Figure S1. Schematic diagram of the three analysis workflows. Workflow A discovers axial length (AL) changepoints in the pooled 6451-eye training dataset using the corrected Akaike Information Criterion (AICc). Workflow B locks those changepoints and applies them to the disjoint single-lens test subgroups (Vivinex, n = 887; SA60AT, n = 821), re-optimizing only the segment-specific constants. Workflow C re-discovers changepoints independently within each test subgroup, serving as the best-achievable benchmark for that lens. No eyes are shared between the training and test datasets.

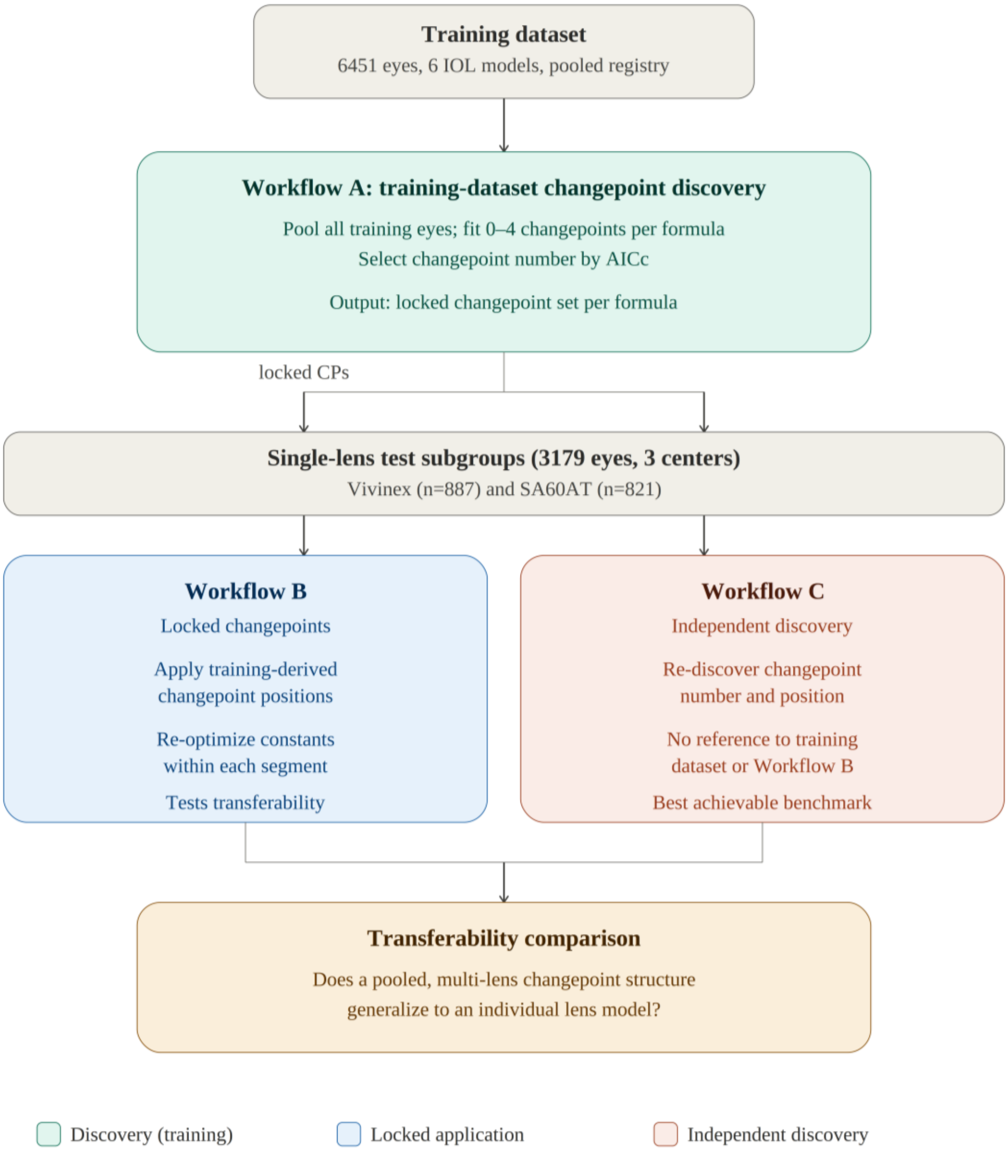

Figure S2. Empirical cumulative distribution function of absolute prediction error for the global versus AL-segmented models in the training dataset, shown for Hoffer Q, Holladay 1 and the classic 3-constant Haigis formula. A leftward shift of the segmented curve indicates a larger proportion of eyes within a given error threshold. D = diopters; PE = prediction error.

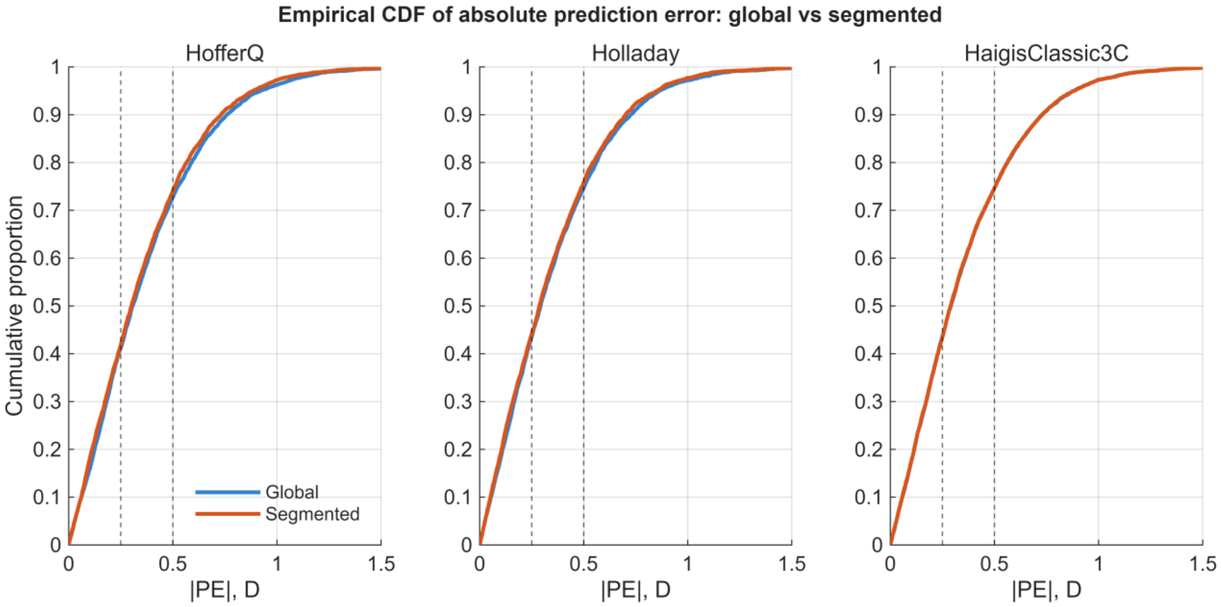

Figure S3. Model-selection profiles showing the Akaike Information Criterion (AIC), the corrected AIC (AICc) and the Bayesian Information Criterion (BIC) as a function of the number of changepoints (0–4) for Hoffer Q, Holladay 1 and the classic 3-constant Haigis formula in the training dataset. The AICc-selected model is marked. For changepoint counts of 3 and 4, the joint minimum segment-size (40 eyes) and minimum segment-width (2.0 mm) constraints limited the search to at most 2 achievable changepoints, so these values coincide with the 2-changepoint solution. AICc selected 1 changepoint for the classic Haigis formula and 2 changepoints for the remaining formulas.

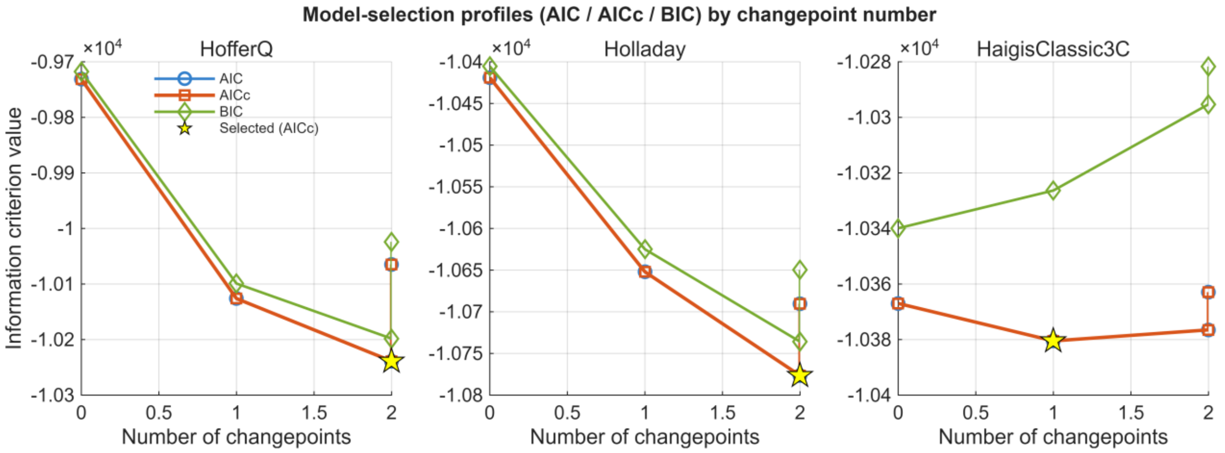

Figure S4. Bland-Altman plot comparing per-eye prediction error from the global model against the AL-segmented model for the Hoffer Q formula in the Vivinex subgroup (Workflow B, n = 887). Each point represents one eye; the solid line indicates the mean difference and the dashed lines the 95% limits of agreement. A mean difference close to zero combined with reduced scatter in the segmented model indicates that segmentation primarily reduces the variance of prediction error rather than shifting its central tendency. D = diopters; PE = prediction error.

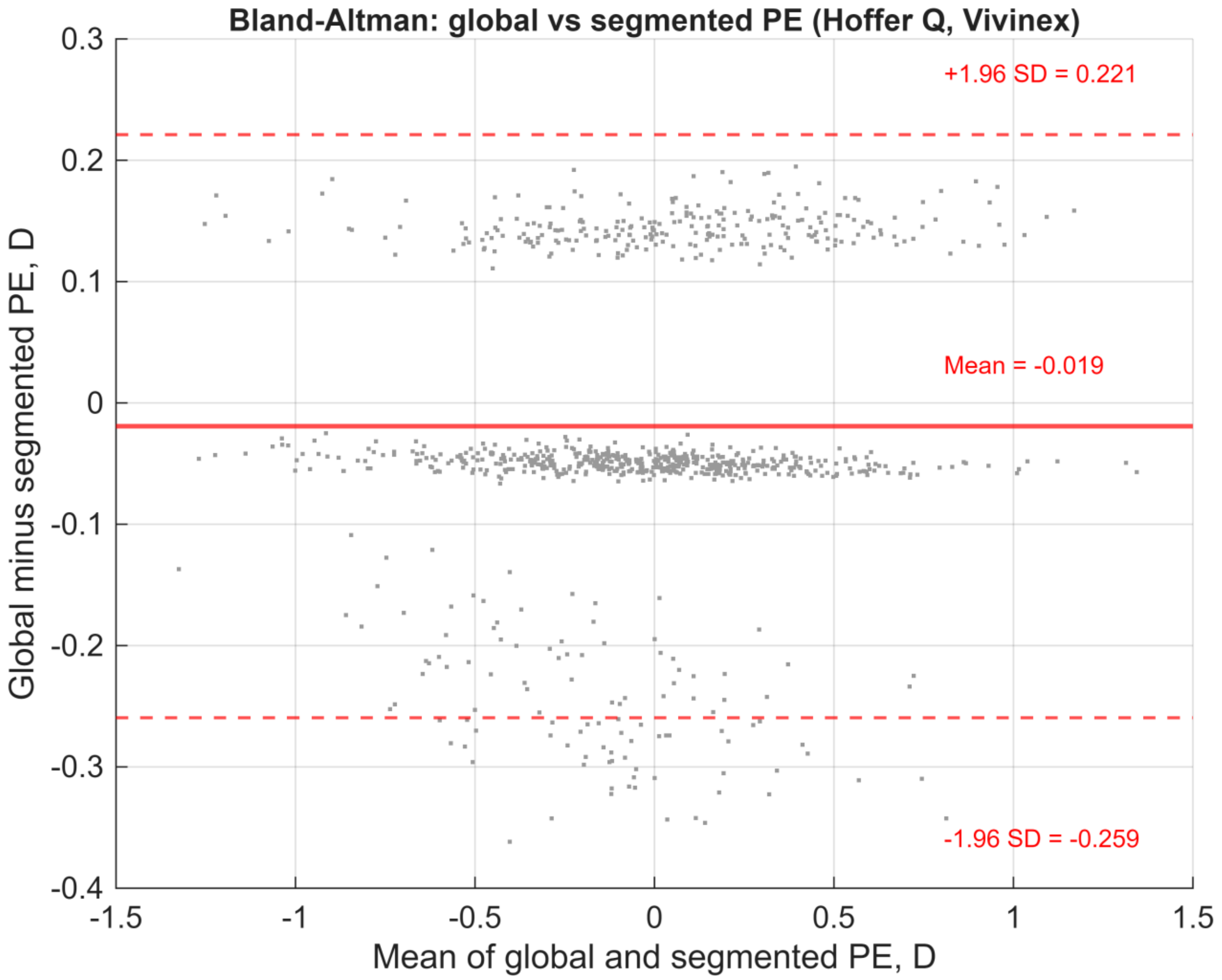

Figure S5. Distribution of axial length in the training dataset (left, n = 6451) and the test dataset (right, n = 3183), in 0.25 mm bins. Red dashed lines mark the AICc-selected Hoffer Q changepoint positions (23.25 and 25.74 mm); grey dotted lines mark the 22.0 mm and 26.0 mm boundaries of the conventional short / normal / long axial length strata. Both datasets are dominated by eyes of normal axial length, so the outer segments are populated by comparatively few eyes.

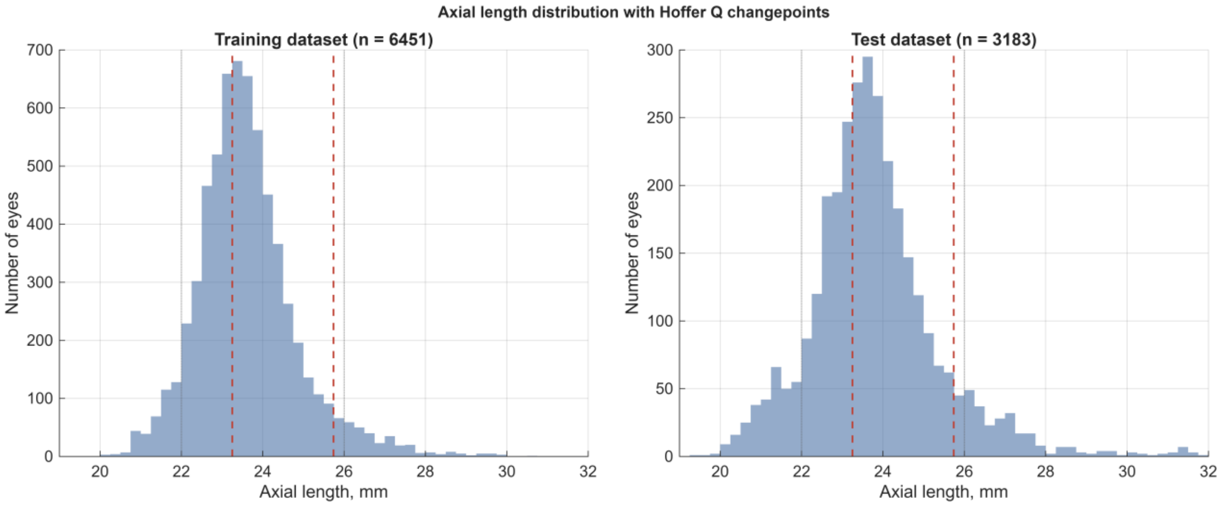

Table S1. Changepoint positions discovered in the pooled training dataset (Workflow A) compared with those discovered independently within each single-lens test subgroup (Workflow C). Deviations are computed over the changepoints that can be paired between the two workflows; where the selected number of changepoints differs, only the first is comparable and this is indicated. A positive deviation denotes a lens-specific changepoint at a longer axial length than its pooled counterpart.

Lens	Formula	Workflow A (mm)	Workflow C (mm)	n paired	Median |dev| (mm)	Shifted up
Vivinex	Classic Haigis	24.49	24.27	1	0.22	0
Vivinex	New-Haigis H1	22.94, 26.41	23.15	1 of 2	0.21	1
Vivinex	New-Haigis H2	22.94, 26.41	23.15	1 of 2	0.21	1
Vivinex	SRK/T	23.25, 25.74	24.37, 26.85	2	1.12	2
Vivinex	Hoffer Q	23.25, 25.74	24.27, 26.85	2	1.07	2
Vivinex	Holladay 1	23.25, 26.41	23.41, 26.15	2	0.21	1
SA60AT	Classic Haigis	24.49	21.58, 25.71	1 of 2	2.91	0
SA60AT	New-Haigis H1	22.94, 26.41	23.61, 25.71	2	0.68	1
SA60AT	New-Haigis H2	22.94, 26.41	23.55, 25.71	2	0.65	1
SA60AT	SRK/T	23.25, 25.74	22.61, 25.71	2	0.33	0
SA60AT	Hoffer Q	23.25, 25.74	21.58, 25.71	2	0.85	0
SA60AT	Holladay 1	23.25, 26.41	23.61, 25.71	2	0.53	1

Across all 20 paired changepoints the median absolute deviation was 0.59 mm (range 0.21 to 2.91 mm). Ten of 20 lens-specific changepoints lay at longer axial lengths than their pooled counterparts and 10 at shorter (two-sided sign test, $p = 1.0$), indicating no systematic directional bias.

Table S2. Sensitivity of Workflow A changepoint discovery to the minimum segment size. Discovery was repeated on the full 6451-eye training dataset with minimum segment sizes of 30, 40 (the value used throughout the manuscript) and 50 eyes. The AICc-selected number of changepoints was unchanged for every formula at every threshold.

Formula	n ≥ 30 eyes	n ≥ 40 eyes	n ≥ 50 eyes	Max shift vs 40 (mm)
Classic Haigis	24.50	24.49	24.49	0.01
New-Haigis H1	22.94, 26.48	22.94, 26.41	22.94, 26.36	0.07
New-Haigis H2	22.94, 26.48	22.94, 26.41	22.94, 26.36	0.07
SRK/T	23.25, 25.78	23.25, 25.74	23.25, 26.36	0.62
Hoffer Q	23.25, 25.78	23.25, 25.74	23.25, 25.70	0.04
Holladay 1	23.25, 26.48	23.25, 26.41	23.25, 26.36	0.07

Changepoint positions were essentially invariant for 5 of the 6 formulas. Only SRK/T showed an appreciable shift, its upper changepoint moving from 25.74 mm to 26.36 mm when the minimum segment size was raised to 50 eyes.

Table S3. Sensitivity of Workflow A changepoint discovery to lens thickness (LT) availability. Discovery on the full 6451-eye training dataset is compared with discovery restricted to the 3510 eyes having a measured and plausible lens thickness. Only the new-Haigis variants use LT, via the corrected axial length; the remaining formulas use raw axial length.

Formula	Full dataset (n = 6451)	LT-available only (n = 3510)	Max shift (mm)
Classic Haigis	24.49	no changepoint selected	—
New-Haigis H1	22.94, 26.41	24.35, 27.68	1.41
New-Haigis H2	22.94, 26.41	27.68 (1 changepoint)	—
SRK/T	23.25, 25.74	23.22, 25.87	0.13
Hoffer Q	23.25, 25.74	22.68, 24.93	0.81
Holladay 1	23.25, 26.41	23.22, 26.51	0.11

Axial length differed slightly but statistically significantly between eyes with and without a usable lens thickness measurement (23.642 ± 1.337 mm versus 23.558 ± 1.085 mm; difference 0.085 mm; t-test $p = 0.006$, Mann-Whitney $p = 0.007$). The magnitude of this difference is negligible relative to the within-group standard deviation of approximately 1.2 mm. Restricting discovery to LT-available eyes left SRK/T and Holladay 1 essentially unchanged but moved the Haigis-family changepoints substantially; Hoffer Q, which uses raw axial length, also shifted by 0.81 mm, indicating that part of this movement reflects the halving of the sample rather than lens thickness itself.
